# Characterization, treatment patterns, and patient-related outcomes of patients with Fragile X syndrome in Germany: final results of the observational EXPLAIN-FXS study

**DOI:** 10.1186/s12888-016-1020-5

**Published:** 2016-09-10

**Authors:** Frank Haessler, Franziska Gaese, Michael Huss, Christoph Kretschmar, Marc Brinkman, Helmut Peters, Samuel Elstner, Michael Colla, David Pittrow

**Affiliations:** 1 Zentrum für Nervenheilkunde, Klinik für Psychiatrie, Neurologie, Psychosomatik und Psychotherapie im Kindes- und Jugendalter, Universitätsmedizin Rostock, Gehlsheimer Str. 20, D-18147 Rostock, Germany; 2 Abt. Psychiatrische Therapie für Menschen mit Geistiger Behinderung, Isar-Amper-Klinikum gGmbH, Klinikum München-Ost, Haar, Germany; 3 Rheinhessen-Fachklinik Mainz, Kinder- und Jugendpsychiatrie, Mainz, Germany; 4 Städt. Krankenhaus Dresden-Neustadt, Zentrum für Kinder- und Jugendmedizin - Sozialpädiatrisches Zentrum, Dresden, Germany; 5 Medizinische Abteilung, Novartis Pharma GmbH, Nürnberg, Germany; 6 Berliner Behandlungszentrum der Abteilung für Psychiatrie, Psychotherapie und Psychosomatik, Evangelisches Krankenhaus Königin Elisabeth Herzberge gGmbH, Berlin, Germany; 7 Experimental and Clinical Research Center, Charité – Campus Berlin Buch & Department of Psychiatry and Psychotherapy, Charité – Campus Mitte, Berlin, Germany; 8 Institut für Klinische Pharmakologie, Medizinische Fakultät, Technische Universität Carl Gustav Carus Dresden, Dresden, Germany; 9 University Medicine, Child and Adolescent Psychiatry, Mainz, Germany

**Keywords:** Fragile X syndrome, Health care, Outcomes, Ambulatory setting, Mental disorders, Caregiver burden, Quality of life

## Abstract

**Background:**

As data on the phenotype, characteristics and management of patients with Fragile X Syndrome (FXS) are limited, we aimed to collect such data in Germany in experienced centres involved in the treatment of such patients.

**Methods:**

EXPLAIN-FXS is a prospective observational (non-interventional) study (registry) performed between April 2013 and January 2016 at 18 sites in Germany. Requirements for patient participation included confirmed diagnosis of FXS by genetic testing (>200 CGG repeats) and written informed consent. Patients were followed for up to 2 years.

**Results:**

Seventy-five patients (84.0 % males, mean age 16.7 ± 14.5 years, ranging from 2 - 82 years) were analysed. The mean 6-item score, determined according to Giangreco (J Pediatr 129:611-614, 1996), was 6.9 ± 2.5 points. At least one neurological finding each was noted in 53 patients (69.7 %). Specifically, ataxia was noted in 5 patients (6.6 %), lack of fine motor skills in 40 patients, (52.6 %), muscle tonus disorder in 4 patients (5.3 %), and other neurological disorders in 39 patients (51.3 %). Spasticity was not noted in any patient.

Seizures were reported in 6 patients (8.1 %), anxiety disorders in 22 patients (30.1 %), depression in 7 patients (9.6 %), ADHD/ADD in 36 patients (49.3 %), impairment of social behavior in 39 patients (53.4 %), and other comorbidities in 23 patients (31.5 %). The mean Aberrant Behaviour Checklist Community Edition (ABC-C) score on behavioral symptoms, obtained in 71 patients at first documentation, was 48.4 ± 27.8 (median 45.0, range 5-115). The mean visual analogue scale (VAS) score, obtained in 59 patients at first documentation, was 84.9 ± 14.6 points (median 90; range 50 – 100).

**Conclusions:**

This report describes the largest cohort of patients with FXS in Europe. The reported observations indicate a substantial burden of disease for patients and their caregivers. Based on these observations, an early expert psychiatric diagnosis is recommended for suspected FXS patients. Further recommendations include multimodal and multi-professional management that is tailored to the individual patient’s needs.

**Trial registration:**

The ClinTrials.gov identifier is NCT01711606. Registered on 18 October 2012.

## Background

Fragile X syndrome (FXS) is among the most common inherited genetic disorders leading to intellectual disability and autism [1]. It is caused by expansion of a cytosine-guanine-guanine (CGG) triplet repeat in the fragile mental retardation 1 (FMR1) gene located on the X chromosome. The presence of more than 200 repeats in the full mutation - compared with 6-44 repeats in normal individuals – is associated with complete or partial absence of the fragile mental retardation protein (FMRP), which regulates neurotransmitter-activated dendritic translation and synaptic plasticity [2]. While both males and females can be affected by FXS, in females, the rates of explicit disease are much lower, and symptoms often milder, due to the inactivation of only one of the two X chromosomes in female cells (all females with FXS are mosaic by definition). A definitive diagnosis can be made via a simple blood sample test and DNA analysis by Southern blot or PCR [3].

Reduced intelligence is a major symptom of FXS, varying from learning difficulties to severe cognitive impairment [4]. Speech, language, and attention deficit occur frequently [5, 6]. Behavioral problems and mood instability often present as the most debilitating aspects of the disease and reduction in these problems are the pivotal focus of drug therapy [7]. Other psychopathological syndromes and disorders are also prevalent: up to 50 % of males with FXS have autistic spectrum disorders [8–10]. Every sixth child with FXS suffers from seizures [11].

Therapeutic options are very limited [12]. The full spectrum of psychotropic drugs (as per label and off-label) is utilized for the treatment of attention deficit disorder, anxiety, hyperactivity, mood swings, anger, depression, seizures, self-injury, and sleep disorders [13, 14].

Further, non-pharmacological therapy such as speech-language therapy or occupational therapy is frequently indicated [15]. In a recent systematic review of 31 intervention studies of individuals with FXS, overall results suggested that a behavioral approach to intervention shows promise [16]. Preliminary experience indicates that assistive technology (i.e. optic sensors such as photocells) generally may be of use to facilitate employment and opportunities of choice [17, 18].

Based on their synaptic mechanisms, specific agents including arbaclofen, ganaxolone, acamprosate, minocycline, as well as the mGluR5 inhibitors AFQ056 (mavoglurant by Novartis) and RG7090 (basimglurant by Roche) have been investigated in FXS [19, 20]. At the time when the present study was planned and initiated, the latter two substances were under investigation in placebo controlled phase IIb/III studies. However, these were discontinued in 2014 due to lack of efficacy in the FXS indication.

In Germany and other European countries, data on the phenotype, characteristics, quality of life, caregiver burden and other aspects of the management of patients with FXS are limited. The current study aimed to fill these gaps. It was initiated as a longitudinal study to investigate changes in psychometric parameters over time.

## Methods

### Patients

Patients (of all ages and both genders) required confirmed diagnosis of FXS by genetic testing (>200 CGG repeats), as well as written informed consent, to participate. No additional genetic testing was required for this study.

Patient data were collected at first documentation and approximately every 6 months thereafter. Patients were to be followed for at least 2 years. As many patients were included at a later stage, the duration of follow-up for these patients was limited.

### Study design

The present study is a prospective observational (non-interventional) study (registry) in Germany. Details of the study protocol have been reported earlier [21]. The study was conducted in accordance with the local laws and regulations [22, 23]. The study identifier at ClinTrials.gov is NCT01711606 (date of registration 18 October 2012).

In 20 % of participating centres, on-site monitoring of the centres was done with source data verification. Physicians received remuneration for participation.

### Setting

Physicians from about 50 centres (hospitals and physician practices), all experienced in the management of FXS, participated in the study. Specialists from other disciplines were also involved, such as child/adolescent psychiatrists, general psychiatrists, or physicians in social paediatric centres.

### Variables

At the first documentation visit, data on the following parameters were documented:

- Demographic data (birth year, gender, height, and weight)
- Availability of prior genetic testing results (to confirm FXS diagnosis)
- Status/medical history of relatives (FXS status in siblings, parents, or other family members; selected comorbidities in father or mother; educational degree of parents). No data on premutation in parents was collected.
- Insurance status (statutory health insurance, private insurance)
- FXS patient history (date of diagnosis and symptoms at diagnosis as measured by the six-item checklist of Giangreco et al. [24]
- Information on autistic-like behaviour (using the checklist by Giangreco et al. [24], specifying characteristics such as tactile defensiveness, perseverative speech, hand flapping, and poor eye contact)
- Noteworthy life events in the past six months (e.g. change of caregiver, relocation, death of relatives, change of school or workplace)
- Educational status or employment status, as appropriate
- Comorbid disorders of the patient (seizures, anxiety, depression, attention deficit disorder, other)
- Neurologic/psychiatric status
- Previous and current medical and non-pharmacological therapy for FXS
- Symptoms Check List (SCL-27) [25, 26]
- Economic parameters: contacts by physicians (various specialties), days of hospitalisation since suspected FXS diagnosis, caregiver’s absence from work
- Results of psychometric testing:
  - ○ IQ testing: Test of adaptive intelligence (Adaptives Intelligenz Diagnostikum, AID) [27], Hamburg-Wechsler intelligence test for children (HAWIK-R/HAWIE-R) [28], Kaufmann Assessment Battery for Children (K-ABC) [29], Snijders Oomen Test [30]
  - ○ Aberrant Behavior Checklist Community Edition (ABC-C) [31, 32], a proxy-completed instrument for rating maladaptive and inappropriate behaviours of individuals with intellectual disabilities
- Quality of life questionnaires (available as paper forms in the study file)
  - ○ EQ-5D (adults) [33] or EQ-5D-Y (children and adolescents) [34], filled out by patient with support from caregiver
  - ○ Eltern Belastungs Inventar (EBI): German version of the Parenting Stress Index (PSI) questionnaire, if caregiver is a family member [35]
  - ○ Nurses’ Observation Scale for In-patient Evaluation (NOSIE), if caregiver is not a family member [36, 37]
  - ○ Short version of the International Classification of Functioning, Disability, and Health (Mini-ICF) rating for psychiatric disorders [38], filled out by the caregiver

At the follow-up visits (approximately every 6 months), information on the following parameters was documented, in the same manner as at first documentation:

- Noteworthy life events (none, change of caregiver, change of residence, divorce or marital separation of parents, death of relative, birth of sibling, starting school, change of workplace, other)
- Educational status and employment status of the patient
- current medical and non-pharmacological therapy
- Economic parameters
- Results of psychometric testing (as at the first documentation visit)
- Quality of life questionnaires (as at the first documentation visit)

Data on patient history and characteristics were retrieved from medical records. Data on quality of life and psychometric assessments were collected on self-administered paper forms which were filled out by the patients and/or their caregivers.

### Statistical analysis

The sample size was based on feasibility aspects. The analysis set consisted of all patients with confirmed FXS diagnosis included in the study. Data were analysed descriptively, using established statistical and epidemiological methods. Continuous variables are reported as median with interquartiles and other percentiles, and as mean with standard deviation (SD), together with minimum and maximum values. Categorical variables were reported as absolute values and relative percentages. Statistical analyses were performed with SAS version 9.2 (Cary, NC, USA).

The authors adhered to the STROBE methodology [39].

## Results

### Patient characteristics

Of the 76 patients who were screened and found eligible, 75 provided data and were included in the analysis. Their characteristics are shown in Table 1. The total number of documented visits was 339 (75.3 % of the 450 maximum total possible visits for the 75 patients).

**Table 1:** Characteristics of patients with FXS and their family members

Number of patients: Characteristic	≤13 years	14-17 years	18+ years	Total (all patients)
	40	11	24	75
Age, years	7.7 ± 3.1	15.3 ± 1.1	32.2 ± 16.4	16.7 ± 14.5
	range, 2-13	range, 14-17	range, 18-82	range, 2-82
Impaired intelligence	38 (95.0 %)	11 (100.0 %)	24 (100.0 %)	73 (97.4 %)
Sex				
Males	35 (87.5 %)	8 (72.7 %)	20 (83.3 %)	63 (84.0 %)
Females	5 (12.5 %)	3 (27.3 %)	4 (16.7 %)	12 (16.0 %)
Siblings				
Males	23 (53.5 %)	10 (58.8 %)	16 (47.1 %)	49 (52.1 %)
Females	20 (46.5 %)	7 (41.2 %)	18 (52.9 %)	45 (47.9 %)
Age of siblings in years	8.9 ± 6.2	15.7 ± 7.9	32.6 ± 21.7	18.4 ± 17.5
	range, 0-26	range, 1-27	range, 1-85	range, 0-85
Siblings with FXS (genetically validated)	14 (32.6 %)	7 (41.2 %)	19 (55.9 %)	40 (42.6 %)
Other family members with FXS	11 (27.5 %)	5 (45.5 %)	17 (70.8 %)	33 (44.0 %)
Main caregiver: patients living with parent(s)	38 (95.0 %)	9 (81.8 %)	15 (62.5 %)	62 (82.7 %)

Values shown are means ± standard deviation or n (%). Impaired intelligence was determined based on school performance, academic achievement, and IQ tests. Attendance at a school for children with special needs was a major criterion for impaired intelligence. Psychometric tests used at the first visit were: the Snijders-Oomen Test (10 patients); the HAWIK/HAWIE-R (8 patients): the Kaufmann Test K-ABC (5 patients); other (not specified) tests (10 patients)

Of these 75 patients, 63 (84.0 %) were males and 12 (16.0 %) were females. The mean age was 16.7 ± 14.5 years, ranging from 2 to 82 years. Patients had siblings with FXS in 17 cases (22.7 %), indicating an extra disease burden on the afflicted families.

The main caregivers were the parents (or a parent) for 62 patients (82.7 %). Notably, of these parents, 45 were married (61.6 %) and 6 were not married but living together (8.2 %).

FXS patients were usually seen by a general practitioner or a paediatrician. (For example, within the 12 months preceding first documentation, patients had seen a GP 3.1 times, and a paediatrician 3.7 times).

The mean 6-item score according to Giangreco (accounting for intelligence impairment, family anamnesis, long narrow face, large or prominent ears, hyperactivity, and autistic features) was 6.9 ± 2.5 points.

Impaired intelligence was determined based on school performance, academic achievement, and IQ tests. Attendance at a school for children with special needs was a major criterion for impaired intelligence. Psychometric tests used at the first documentation were the Snijders Oomen-Test (10 patients); the HAWIK/HAWIE (8 patients); the Kaufman Test K-ABC (5 patients); other (not specified) tests (10 patients).

For the calculation of the Giangreco index (*n* = 75 patients), 65 patients had an IQ value < 70 points; 8 patients an IQ value of 70 to 85 points; and 2 patients > 85 points. Overall, 73 patients (97.3 %) were deemed to have impaired intelligence.

Of the 75 patients, 53 suffered from one or more neurological signs (69.7 %; Table 2). Specifically, ataxia was noted in 5 patients (6.6 %), lack of fine motor skills in 40 patients (52.6 %), muscle tonus disorder in 4 patients (5.3 %), and others in 39 patients (51.3 %). Spasticity, defined as spastic paresis or occurrence of pyramidal signs, was not noted in any patient.

**Table 2:** Neurological findings

Finding	≤13 years (*n* = 40)	14-17 years (*n* = 11)	18+ years (*n* = 24)	Total (all patients, *n* = 75)
	*n* (%)	*n* (%)	*n* (%)	*n* (%)
At least one of the following	33 (82.5)	9 (81.8)	11 (45.8)	53 (69.7)
Ataxia	4 (10.0)	0	1 (4.2)	5 (6.6)
Spasticity	0	0	0	0
Impaired fine motor skills	26 (65.0)	9 (81.8)	5 (20.8)	40 (52.6)
Muscle tonus disorders	3 (7.5)	1 (9.1)	0	4 (5.3)
Others	24 (60.0)	6 (54.5)	9 (37.5)	39 (51.3)

These conditions were established by treating physicians

Spasticity was defined as spastic paresis or occurrence of pyramidal signs

Multiple answers were possible. “Other” neurological findings were not further specified by documenting physicians

### Comorbidities and treatment

The prevalence of seizures and mental disorders was high (Table 3). Seizures were reported in 6 patients (8.1 %), anxiety disorders in 22 patients (30.1 %), depression in 7 patients (9.6 %), ADHD/ADD in 36 patients (49.3 %), and impairment of social behavior in 39 patients (53.4 %). Other comorbidities were noted in 23 patients (31.5 %), of which the following were seen in more than 1 individual: obesity (5 patients), sleep disorder (4), foot deformity (3), autism and autism spectrum disorder (4), and increased infection susceptibility (2). Comorbidities were treated with a variety of medications which are detailed in Table 4. The most frequently noted drugs were amphetamines and methylphenidate, for the indications of ADHD and behaviour disorders.

**Table 3:** Prevalence of seizures and mental disorders

Disorder	≤13 years (*n* = 40)	14-17 years (*n* = 11)	18+ years (*n* = 24)	Total (all patients, *n* = 75)
	*n* (%)	*n* (%)	*n* (%)	*n* (%)
Seizures	5 (12.8)	0	1 (4.2)	6 (8.1)
Anxiety disorders	12 (30.8)	3 (27.3)	7 (30.4)	22 (30.1)
Depression	3 (7.7)	1 (9.1)	3 (13.0)	7 (9.6)
ADHD/ADD	24 (61.5)	7 (63.6)	5 (21.7)	36 (49.3)
Impairment of social behaviour	22 (56.4)	6 (54.5)	11 (47.8)	39 (53.6)
Other	11 (28.2)	4 (36.4)	8 (34.8)	23 (31.5)
Suicidality	0	0	0	0

These conditions were established by treating physicians

“Other” disorders were not further specified by documenting physicians

**Table 4:** Drug treatment of seizures and mental disorders in the 12 months prior to first documentation

Comorbidity category (n of afflicted patients)	Substance	Patient number	≤13 years (*n* = 40)	14-17 years (*n* = 11)	18+ years (*n* = 24)	Total (all patients, *n* = 75)
			*n* (%)	*n* (%)	*n* (%)	*n* (%)
ADH (36)	Amphetamine		9 (37.5)	4 (57.1)	1 (20.0)	13 (17.1)
	Atomoxetine, Desipramine, etc.		0	1 (14.3)	0	2 (2.6)
	Methylphenidate		9 (37.5)	3 (42.9)	1 (20.0)	14 (18.4)
	Risperidone, Quetiapine, etc.		3 (12.5)	1 (14.3)	0	6 (7.9)
	Other		11 (45.8)	2 (28.6)	1 (20.0)	14 (18.4)
Anxiety (22)	Fluoxetine, Fluvoxamine, etc.		5 (41.7)	2 (66.7)	2 (28.6)	9 (11.8)
	Amitriptyline, Imipramine, etc.		0	0	0	
	Diazepam, Lorazepam, etc.		0	0	0	
	Risperidone, Clozapine, etc.		0	0	2 (28.6)	2 (2.6)
	Other		6 (50.0)	2 (66.7)	1 (14.3)	9 (11.8)
Conduct disorders (39)	Methylphenidate, Amphetamine, etc.		8 (36.4)	2 (33.3)	2 (18.2)	12 (15.8)
	Pipamperone, Melperone, etc.		0	0	0	1 (1.3)
	Risperidone, Clozapine, etc.		5 (22.7)	0	2 (18.2)	10 (13.2)
	Other		14 (63.6)	4 (66.7)	4 (36.4)	23 (30.3)
Depression (7)	Fluvoxamine, Citalopram, etc.		2 (66.7)	1 (100.0)	1 (33.3)	4 (5.3)
	Venlafaxine, Bupropion, etc.		0	1 (100.0)	0	
	Lithium			1 (100.0)		
	Other		1 (33.3)	0	0	1 (1.3)
Seizures (6)	Valproate		1 (20.0)	0	1 (100.0)	2 (2.6)
	Other		3 (60.0)	0	1 (100.0)	6 (7.9)

Values indicate patient numbers (n) and, in brackets, the percentage of afflicted patients in the respective age group

Among non-pharmacological therapies, the following were noted at first documentation, in descending frequency: speech-language therapy, occupational therapy, therapeutic pedagogy, osteopathy, music therapy, sociotherapy, animal assisted therapy, and various other therapies (Table 5).

**Table 5:** Non-pharmacological therapy (number of appointments) in the 12 months prior to inclusion

Therapy	≤13 years (*n* = 40)		14-18 years (*n* = 11)		18+ years (*n* = 24)		Total (*n* = 75)	
	*n*	mean	*n*	mean	*n*	mean	*n*	mean
Psychotherapy	9	5.1 ± 13.2	0	-	11	2.6 ± 7.5	20	3.8 ± 10.2
Occupational therapy	23	30.6 ± 17.9	9	25.9 ± 17.6	11	-	43	21.8 ± 19.9
Speech therapy	32	30.8 ± 17.2	4	37.0 ± 29.1	9	1.1 ± 3.3	45	25.4 ± 20.5
Sociotherapy	8	5.0 ± 14.1	1	30.0	9	-	18	3.9 ± 11.4
Therapeutic pedagogy	18	21.4 ± 20.4	1	20.0	10	4.0 ± 12.6	29	15.3 ± 19.4
Music therapy	10	14.0 ± 19.0	1	40.0	9	0	20	9.0 ± 16.5
Osteopathy	10	0.9 ± 1.5	1	2.0	10	0.3 ± 0.9	21	0.7 ± 1.3
Animal-assisted therapy	3	0.0 ± 0.0	0	-	4	-	7	0.0 ± 0.0
Other	29	39.7 ± 35.6	4	15.3 ± 18.9	10	4.0 ± 12.6	43	29.1 ± 33.9

### Life events at first documentation

Relevant life events included change of reference person to the patient (2 cases, 2.6 %), moving home (5 cases, 6.6 %), separation of parents (3 cases, 3.9 %), death of a close person (2 cases, 2.6 %), starting school/work (1 patient, 1.3 %), and other events (9 patients, 11.7 %). No such relevant life events occurred in 59 patients (77.6 %).

### School type

Fifty one patients (77.3 %) had been attending school, and 33 of these patients were currently attending school. Mean duration of school education was 7.7 ± 3.6 years (range 1- 15). Of these, 32 children and adolescents (62.7 % of those attending a school) were in a school for the intellectually disabled, 14 (27.5 %) attended a similar school type for learning disabled students (“Förderschule”), 7 (13.7 %) attended a regular school with integration measures, 2 (3.9 %) attended a regular school, and 3 (4.9 %) attended a different school type (none of the above).

### Psychometric and sociometric assessments

The 27-item symptom checklist mean score, calculated in 54 patients at first documentation, was 4.7 ± 3.1 points.

The patients had taken various IQ tests prior to first documentation or took these during the course of the study.

The Aberrant Behaviour Checklist Community Edition (ABC-C) score, obtained in 71 patients at first documentation, was 48.4 ± 27.8 (median, 45.0; range, 5-115; Table 6). At follow-up 1 (in 60 patients) the score was 38.6 ± 23.4 points; at follow-up 2 (in 48 patients), it was 42.0 ± 25.1 points; at follow-up 3 (in 41 patients), it was 43.0 ± 23.1 points; and at follow-up 4 (in 27 patients), it was 44.3 ± 22.7 points.

**Table 6:** ABC Community score at first documentation

	≤13 years (*n* = 40)	14-17 years (*n* = 11)	18+ years (*n* = 24)	Total (all patients, *n* = 75)
Score/ subscores				
Total score	55.4 ± 29.7	48.5 ± 23.0	36.4 ± 23.1	48.4 ± 27.8
Irritability	18.4 ± 11.8	15.4 ± 11.6	9.1 ± 7.6	15.0 ± 11.3
Lethargy	7.7 ± 6.2	6.4 ± 5.1	6.9 ± 7.1	7.2 ± 6.3
Stereotypic behaviour	6.6 ± 4.9	5.1 ± 4.8	3.4 ± 3.3	5.4 ± 4.6
Hyperactivity	13.5 ± 7.7	8.7 ± 5.7	7.4 ± 6.5	10.9 ± 7.5
Inappropriate speech	4.1 ± 3.4	5.9 ± 3.4	4.1 ± 3.7	4.4 ± 3.5
Social avoidance	3.6 ± 3.0	6.0 ± 3.3	3.9 ± 3.6	4.0 ± 3.3

The parenting stress index (PSI) total score, obtained in 64 patients, was 74.6 ± 12.1 (median, 85.0; range, 47 to 85). In the child domain, the mean score was 78.5 ± 10.0 and in the parent domain, it was 65.6 ± 13.2. Overall, patients reported a low/normal stress level in 6 cases (9.4 %), a high stress level in 21 cases (32.8 %), and a very high stress level in 37 cases (57.8 %). Children reported a low/normal stress level in 2 cases (3.1 %), a high stress level in 14 cases (21.9 %), and a very high stress level in 48 cases (75.0 %). In parents, the stress level was lower, and low/normal stress levels were reported by 25 respondents (39.1 %), high stress levels by 22 respondents (34.4 %), and very high stress levels by 50 respondents (78.1 %).

The Nurses’ Observational Scale for Inpatient Evaluation (NOSIE) mean index value, based on answers from 28 patients at first documentation, was 151.5 ± 0.5 (median, 151.1; range, 151 -153).

On the mini-ICF, the average value at first documentation, obtained in 73 patients, was 2.2 ± 0.8 (median, 2.2; range, 0 to 4).

### EuroQol 5D-3 L

The EQ-5D index in adults at first documentation, obtained in 41 patients, was 0.7 ± 0.3 points (median, 0.7; range, 0-1). The EQ-5D index in children at first documentation, obtained in 22 patients, was 0.7 ± 0.3 points (median, 0.8; range, 0-1). The EQ-5D time trade-off value in adults at first documentation, obtained in 22 patients, was 0.8 ± 0.3 points (median, 0.9; range, 0-1). The EQ-5D time trade-off value in children at first documentation, obtained in 41 patients, was 0.7 ± 0.3 points (median, 0.9; range, 0-1). On the EQ-5D visual analogue scale (VAS), the mean value at first documentation, obtained in 59 patients, was 84.9 ± 14.6 points (median, 90; range, 50 - 100).

## Discussion

The EXPLAIN study is the largest and the only longitudinal European observational study investigating clinical characteristics and caregiver situation of patients with FXS. It documents 75 individuals in a wide age range, from 2 to 82 years. As expected, the prevalence of neurological findings and other comorbidities was very high. Almost every patient in our study had a relevant mental or neurological disorder which required pharmacological or non-pharmacological treatment. In younger patients, externalizing symptoms prevailed; in older patients, internalizing symptoms prevailed.

The only systematic recording of data on patients with FXS is currently taking place in the USA, in the context of individual projects, namely by the Fragile X Clinical & Research Consortium (FXCRC), with its FORWARD Registry and Database, and by our Fragile X World Surveys (twice per year, also supported by the U.S. Centers for Disease Control) [14, 15, 40].

The majority of documented patients in the present study were children and adolescents; only 29 were adults. Thus, it is understandable that most patients were still living with their parents, who mostly were the main caregivers. This indicates solid family structures, and might be a finding based on patient selection. The proxy information collected in this study can be considered to be “first-hand” and very reliable.

In light of the common social and behavior problems linked with FXS, an important tool for our study was the ABC-C questionnaire. It is a sensitive instrument for rating maladaptive and inappropriate behaviors in patients with intellectual disabilities. In patients with FXS, it has also been used as an endpoint in clinical studies (in the original or a modified version) [41–43]. For example, in the mavoglurant studies, mean total scores at first documentation were, across the placebo and treatment groups, between 40.0 and 50.6 points in adults, and between 48.3 and 58.5 points in adolescents. The corresponding scores in our study were substantially lower in adults (36.4 points), and similar in adolescents (48.5 points). During the study, we found small improvements in the ABC-C score which remained constant over time. This might represent a study artefact or a nonspecific benefit of study participation.

The study shows a high burden of comorbidities as well as mental symptoms. The rates of mental health symptoms, at least in children and adolescents, were higher than the prevalence of such symptoms in the general population of individuals with intellectual disability in this age range. The prevalence of mental health symptoms in the general population of individuals with intellectual disability has been documented as follows: 24 % with impaired social behavior; 13 % with emotional problems; and 26 % with hyperactivity [44]. In EXPLAIN, the prevalences were 53.6 %, 39.7 %, and 49.3 %, respectively. As autistic behaviors were not specifically assessed in EXPLAIN, no conclusions can be drawn about them. However, overall, 42 % of parents and other caregivers reported such symptoms in their patients without describing them in detail. This prevalence is comparable with international studies reporting a prevalence of autistic symptoms between 25 and 47 % [45].

Compared with another study in a large US American cohort (89 women and 239 men aged between 22 and 64 years) [46], affective symptoms were less frequent in the adults (71.7 % versus 43.4 %). Hyperactivity in this age range occurred in only 21.7 %. Conversely, Hartley et al. described hyperactivity in 64.3 % of adults with FXS [46].

It was an unexpected finding that the comorbid mental health symptoms and diseases usually were treated with medications. Usually the prescription of psychopharmacological drugs in individuals with impaired intelligence is between 21 and 45 % [47]. The high prescription prevalence is probably an indicator of the severity of the individual disorder. Usually, for patients with FXS, findings from other neurodevelopmental disorders such as autism or other forms of intellectual disability are extrapolated for therapy planning [48–50]. The FXCRC has highlighted that this patient group is sensitive to psychotropic medication, and recommends low doses and cautious titration [51].

In terms of quality of life assessment, as expected, patients with FXS had limitations in terms of self-care and usual activities, but also anxiety/depression. Compared with the German reference population [52], patients with FXS reported similar values for general health (visual analog scale). The EQ-5D questionnaire is widely used in studies with adults as a source of generic health-related quality of life information and utility weights to inform resource allocation decisions. Despite methodological limitations, the EQ-5D in its “youth” form is suitable for the same aim in children [34, 53]. On the EQ-5D scale, the EQ-5D time trade off (TTO) consists of a hypothetical trade-off between a shorter lifespan and a healthier quality of life. The respective reference score for the FXS population was calculated as described by Greiner et al. [33] The utility index value (0.8) was high, but showed substantial variation between patients. It remained relatively constant across the follow-up visits.

As a limitation to this study, a number of instruments which were used in other FXS studies were not applied in our study. For example, the mavoglurant studies had applied, among others, the SRS scale to identify the presence of autistic social impairment; the TEA-Ch to test every-day attention and inhibition in children; or the widely used Clinical Global Impression (CGI) scale to assess treatment response in psychiatric patients [42]. This study might be prone to selection bias: [54] participating centres might represent a positive selection with particular interest in FXS (and research questions). Participating patients (or their caregivers) might be more likely than others to understand the disease and to contribute to the optimization of management. Finally, we did not collect information on associated non-neurological medical problems which are known to be frequent among patients with FXS (such as otitis media, gastrointestinal problems and obstructive sleep apnea), and which increase the propensity for many behavioural problems [55].

## Conclusion

In conclusion, the findings in EXPLAIN-FXS confirm the substantial burden of psychiatric and mental problems in patients with FXS in all age groups. In these patients, an early expert psychiatric diagnosis should be initiated. The resulting management should be multimodal and multi-professional and should be tailored to the individual patient’s needs.

## Abbreviations

ABC-C: Aberrant behaviour checklist (Community Edition)
ADD: Attention deficit disorder
ADHD: Attention deficit hyperactivity disorder
BfArM: Bundesinstitut für Arzneimittel und Medizinprodukte
BMI: Body mass index
CGG: Cytosine guanine guanine (triplet)
CRO: Clinical Research Organisation
EBI: Eltern-belastungs-inventar
EQ-5D: EuroQol 5 Dimensions
EQ-5D-Y: EuroQol 5 Dimensions Youth
FMR: Fragile X mental retardation
FMRP: Fragile X mental retardation protein
FSA: Freiwillige Selbstkontrolle für die Arzneimittelindustrie
FU: Follow-up
FXS: Fragile X Syndrome
ICF: International Classification of Functioning, Disability and Health
IQ: Intelligence quotient
NIS: Non-interventional study
NOSIE: Nurses’ observation scale for inpatient evaluation
PSI: Parental stress index
SCL: Symptom checklist
SDV: Source document verification
TTO: Time to trade off
VAS: Visual analog scale
VfA: Verband Forschender Arzneimittelhersteller
